# An Incarcerated Colon Inguinal Hernia That Perforated into the Scrotum and Exhibited an Air-Fluid Level

**DOI:** 10.1155/2015/105183

**Published:** 2015-05-13

**Authors:** Seisuke Ota, Toshio Noguchi, Tomoya Takao, Takumi Sakamoto, Yuichiro Kanie, Ken-ichi Omae, Shunji Fujie, Yoshiaki Kanaya, Akinori Kasahara, Tadashi Matsumra

**Affiliations:** ^1^Department of Internal Medicine, Himeji St. Mary's Hospital, 650 Nibuno, Himeji 670-0801, Japan; ^2^Department of Surgery, Himeji St. Mary's Hospital, 650 Nibuno, Himeji 670-0801, Japan; ^3^Department of Radiology, Himeji St. Mary's Hospital, 650 Nibuno, Himeji 670-0801, Japan

## Abstract

There are few reports of a transverse colon inguinal hernia; furthermore, an inguinal hernia perforating the scrotum is rare. Here we report the case of a 79-year-old man who died after developing an incarcerated colon inguinal hernia that perforated the scrotum and exhibited an air-fluid level. The patient was referred to our hospital in November 2011 with a complaint of inability to move. Physical examination revealed an abnormally enlarged left scrotum and cold extremities. He reported a history of gastric cancer that was surgically treated more than 30 years ago. His white blood cell count and C-reactive protein level were elevated. Abdominal and inguinal computed tomography revealed that his transverse colon was incarcerated in the left inguinal canal. Free air and air-fluid level were observed around the transverse colon, suggestive of a perforation. The patient and his family refused any surgical intervention; therefore, he was treated with sultamicillin tosilate hydrate and cefotiam hydrochloride. However, he succumbed to panperitonitis 19 days after admission. The findings from this case indicate that the transverse colon can perforate into an inguinal hernia sac.

## 1. Introduction

Inguinal hernia is a common problem in adults, and obstruction and strangulation are the most serious complications [1]. There are two types of inguinal hernia, direct and indirect, which are defined by their relationship to the inferior epigastric vessels. Direct inguinal hernias occur medial to the inferior epigastric vessels when abdominal structures herniate through a weak spot in the fascia of the posterior wall of the inguinal canal, which is formed by the transversalis fascia. Indirect inguinal hernias occur when abdominal structures protrude through the deep inguinal ring, lateral to the inferior epigastric vessels; this may be caused by the failure of embryonic closure of the processus vaginalis [2].

Hernias are more common in men than in women. Men are eight times more likely to develop a hernia and 20 times more likely to require hernia repair [3]. The lifetime risk of developing inguinal and femoral hernias is approximately 25% in men and less than 5% in women. In one review, the median age at presentation was 60–79 years for women and 50–69 years for men [4].

An inguinal hernia rarely perforates into the scrotum. Here we report a rare case of an inguinal incarcerated hernia that perforated the scrotum and exhibited an air-fluid level.

## 2. Case Presentation

A 79-year-old man was referred to our hospital in November 2011 with a complaint of inability to move. His body temperature was 37.7°C, blood pressure was 103/58 mm Hg, peripheral oxygen hemoglobin saturation was 95%, and respiration rate was 25 breaths/min. Physical examination revealed an abnormally large left scrotum and cold extremities. He reported a history of gastric cancer, for which he had undergone surgery more than 30 years ago. His white blood cell count was 9600/*μ*L (92.0% polymorphonuclear neutrophils), and his C-reactive protein level was 34.5 mg/dL.

**Figure 1:**
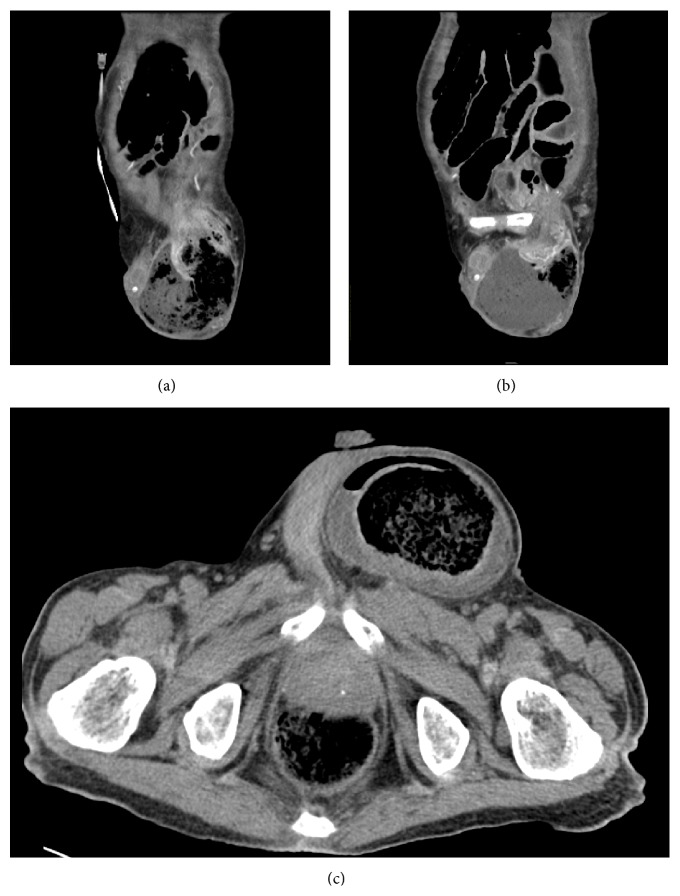
The transverse colon was incarcerated in the left inguinal canal (a, b). Free air and air-fluid level were observed around the transverse colon, suggestive of a perforation (c).

Abdominal and inguinal computed tomography (CT) revealed an inguinal hernia that had perforated into the left scrotum and exhibited an air-fluid level (Figure 1). These CT findings were compatible with those for direct hernia.

The patient did not have a wife or children, and he and his younger sister refused surgical intervention because he did not wish to live longer, although he had no other health problems. He was treated with sultamicillin tosilate hydrate and cefotiam hydrochloride. However, abdominal CT performed on the 5th day revealed progression to panperitonitis, and he succumbed to this condition 19 days after admission.

## 3. Discussion

Inguinal and femoral hernias exhibit various clinical features that range from an innocuous bulge in the groin region on routine physical examination (with or without pain) to life-threatening bowel strangulation. Incarcerated or strangulated hernias can present as an acute mechanical intestinal obstruction without obvious symptoms or signs of a groin hernia, particularly if the patient is obese. Abdominal CT is the most powerful diagnostic aid. Emergency surgical repair is indicated for those patients who develop complications, including bowel obstruction and bowel perforation, related to inguinal or femoral hernia. The overall risk of incarceration and strangulation is low, with an estimated incidence of 0.3%–3% per year [5–9].

There are a few reports on a transverse colon structure in an inguinal hernia [10, 11]. A case of enteroscrotal fistula was reported in a Ghanaian adult [12]. In addition, a case of a neglected, strangulated inguinal hernia that presented as a scrotal fecal fistula in a middle-aged male was also reported [13]; the authors claimed that this was the first such case reported in an adult. Seven other cases documented in the literature were all pediatric cases [14–19]. Three cases of incarcerated appendix have been reported, of which one was of gangrene in the ascending colon and the proximal part of the transverse colon, with the tip of the appendix within the hernial sac [20]. However, we found no reports of transverse colon perforation into the scrotum.

Unfortunately, we could not perform life-saving surgery for this patient because he and his family refused the option, even though we explained all the benefits and risks. It would be interesting to know the outcome with the suggested surgical approach. The present case shows that an incarcerated inguinal hernia of the colon can perforate into the scrotum and exhibit an air-fluid level.

## 4. Conclusions

The findings from this case indicate that the transverse colon can perforate into an inguinal hernia sac.
